# Role of autophagy in K-RAS- and B-RAF-driven lung cancers

**DOI:** 10.1186/2049-3002-2-S1-O11

**Published:** 2014-05-28

**Authors:** Eileen White

**Affiliations:** 1 Rutgers Cancer Institute of New Jersey, New Brunswick, NJ 08903, USA; 2 Department of Molecular Biology and Biochemistry, Rutgers University, Piscataway, NJ, 08854, USA

## 

Autophagy degrades and recycles proteins and organelles to support metabolism and survival starvation. Oncogenic RAS upregulates autophagy required for mitochondrial function, stress survival, and engrafted tumor growth. We deleted an essential autophagy gene, autophagy-related-7 (*Atg7*), concurrently with *Kras^G12D^* activation with or without intact *Trp53* in two mouse models for non-small-cell lung cancer (NSCLC). In both models, *Atg7* deficiency caused tumor cells to accumulate dysfunctional mitochondria, and acquire metabolic, growth and survival defects associated with reduction in tumor burden. Importantly, *Atg7* deficiency altered the fate of *Kras^G12D^*-induced carcinomas to that of oncocytomas, rare, predominantly benign tumors characterized by the accumulation of defective mitochondria. Surprisingly, lipid accumulation was observed in *Atg7*-deficient tumors only when *Trp53* was deleted. *Atg7*-deficient tumor-derived cell lines (TDCLs) had compromised starvation survival and formed lipidic cysts instead of tumors, suggesting defective utilization of lipid stores. *Atg7* deficiency reduced fatty acid oxidation (FAO) and increased sensitivity to FAO inhibition, indicating that with *Trp53* loss, RAS-driven tumors require autophagy for mitochondrial function and lipid catabolism. Thus, autophagy is required for carcinoma fate, and cancers require autophagy for distinct roles in metabolism that are oncogene- and tumor suppressor gene-specific. To test the role of autophagy in oncogenic signaling pathways downstream of RAS, *Atg7* was deleted in a mouse model of *BRAF^V600E^*-induced lung cancer in the presence or absence of the tumor suppressor *Trp53*. *Atg7* deletion initially induced oxidative stress and accelerated tumor cell proliferation in a manner indistinguishable from *Nrf2* ablation.

Compound deletion of *Atg7* and *Nrf2* had no additive effect suggesting that both genes modulate tumorigenesis by regulating oxidative stress, revealing a potential mechanism of autophagy-mediated tumor suppression. At later stages of tumorigenesis, *Atg7* deficiency resulted in an accumulation of defective mitochondria, proliferative defects, reduced tumor burden, conversion of adenomas and adenocarcinomas to oncocytomas, and increased mouse lifespan. Autophagy-defective tumor-derived cell lines were defective in their ability to respire, survive starvation and were glutamine-dependent, suggesting that autophagy-supplied substrates from protein degradation sustains *Braf^V600E^*-tumor growth and metabolism.

